# The occurrence and development of radiation-induced lung injury after interstitial brachytherapy and stereotactic radiotherapy in SD rats

**DOI:** 10.1186/s12950-023-00348-9

**Published:** 2023-07-10

**Authors:** Zhuo Chen, Bin Wang, Zhouxue Wu, Hua Xiao, Yang Yang, Junying Fan, Yingjiang Gu, Chuan Chen, Jingbo Wu

**Affiliations:** 1 Department of Oncology, Daping Hospital, Army Medical University, 10 Changjiang Branch Road, Yuzhong District, Chongqing, 400042 China; 2 Department of Oncology, Affiliated Hospital of Southwest Medical University, No.25 Taiping Street, Jiangyang District, Luzhou, 646099 Sichuan China; 3 Department of Oncology, the Seventh People’s Hospital of Chongqing (Affiliated Central Hospital of Chongqing University of Technology), Banan District Lijiatuo Industry Federation No.1 Village, Chongqing, 401320 China; 4 Department of Neurosurgery, Affiliated Hospital of Traditional Chinese Medicine of Southwest Medical University, Longmatan District, No. 182 Chunhui Road, Luzhou, 646099 Sichuan China; 5 Key Laboratory of Nuclear Medicine and Molecular Imaging, Changzhi, 046099 Sichuan China

**Keywords:** Interstitial brachytherapy, Stereotactic body radiotherapy, Radiation-induced lung injury, Lung function, Inflammatory factor

## Abstract

**Background:**

To compare the severity of radiation-induced lung injury (RILI) after the right lung of SD rats received interstitial brachytherapy and stereotactic radiotherapy (SBRT).

**Methods:**

RILI rat model was established using interstitial brachytherapy and SBRT methods, respectively. CT scan was performed to analyze the lung volume and the CT value difference between the left and right lungs in rats. Then the lung tissues were analyzed through H&E staining, peripheral blood was extracted to detect the expression levels of serum inflammatory cytokines, pro-fibrotic cytokines, and fibrotic-inhibiting cytokines by ELISA.

**Results:**

The difference between right and left lung CT values was significantly elevated in the SBRT group when compared with the control group and the interstitial brachytherapy group (*P* < 0.05). The IFN-γ expression in the interstitial brachytherapy group was significantly different from that in the SBRT group at week 1, 4, 8 and 16. Besides, the expressions of IL-2, IL-6 and IL-10 in SBRT group were significantly higher than that of interstitial brachytherapy group (*P* < 0.05). The TGF-β expression in interstitial brachytherapy group reached its peak with the increase of time from week 1 to week 16, and it was significantly lower than SBRT group (*P* < 0.05). The mortality rate in the SBRT group was 16.7%, which was significantly higher than that in the interstitial brachytherapy group.

**Conclusion:**

The treatment method of interstitial brachytherapy is considered as an effective and safe tool by reducing the side effects of radiotherapy and increasing the radiation dose of radiotherapy.

## Background

Lung is one of the most sensitive tissues to radiation, which limits the dose and therapeutic effect of radiotherapy [1]. Approximately 10–30% of patients present radiation-induced lung injury (RILI) after receiving chest radiotherapy, seriously affecting patients' cardiopulmonary function and life quality [2]. The main clinical manifestations of RILI include dry cough, shortness of breath, chest pain, fever, and even respiratory failure and death, which are not only associated with tumor site, tumor type, size and pathology, but also with lung function, the dose of radiotherapy, chemotherapy application, targeted therapy and immunotherapy [3, 4]. The course of RILI usually divides into early pulmonary toxicity which occurs within hours to days after radiotherapy [5], and late pulmonary toxicity which occurs months to years after treatment, including pulmonary tissue fibrosis, necrosis and atrophy [6]. As one of the main anti-tumor treatments in China at present, radiotherapy can rapidly relieve tumor-related symptoms and prolong the survival cycle of patients, however, the radiation dose is limited by the radiation tolerance of the surrounding normal tissues [7]. Therefore, precision radiotherapy such as stereotactic body radiotherapy has been widely applied in clinics for its great advantages such as good effect, rapid recovery and less trauma in recent years, but it still cannot achieve the improvement of radiotherapy dose.

The underlying mechanism of RILI is very complicated, it is involved the injury or apoptosis of a large number of normal alveolar epithelial cells and vascular endothelial cells induced by radiation therapy, thus releasing a wide range of inflammatory mediators and contributing to the occurrence and development of RILI [8–10]. The activation of alveolar macrophages is generally considered to play an important role in RILI, which is largely responsible for the persistent cytokine cascade and chronic inflammatory state leading to early and late lung injury [11]. The occurrence of DNA double-strand breaks, cytoplasmic or organelle damage in patients with malignant tumors after several hours or days post-radiotherapy triggers intracellular signal transduction, thus activating immune cells to the secretory large profile of inflammatory mediators such as interleukin (IL)-2, IL-6, IL-10, interferon (IFN)-γ, and transforming growth factor (TGF)-β [12, 13]. They all belong to the RILI-related cytokines with different cell origins and functions.

Multiple risk factors such as inflammation, oxidative stress and mitochondrial dysfunction participate in the progression of RILI [14–16]. The specific pathogenesis of RILI is difficult to determine, and there is a lack of timely and effective drug intervention. Besides, once RILI occurs, powerful supportive treatment such as mechanical ventilation, steroids and antibiotics is needed. However, even with supportive therapy, RILI is still potentially fatal [17]. Interstitial brachytherapy has the feature of rapidly dropping the radiation dose around the tumor to the lowest level, which is a more reasonable alternative for the unresectable tumor with complex location structures, recurrence or metastasis. Moreover, interstitial brachytherapy also has several advantages such as convenient operation, good tolerance, low cost, slight damage to normal tissue, and fewer side effects [18]. It is reported that the therapeutic effect of interstitial brachytherapy to increase radiotherapy dose can achieve a similar effect of radical surgery, considered as “proton therapy” for patients with a poor economic capacity [19].

In this study, radiation pneumonia SD rats were established, and we identified for the first time that the radiological manifestations of RILI after interstitial brachytherapy are less severe, with a slight impact on lung function and lung volume. This study provides novel insights for patients with malignant tumors treated with interstitial brachytherapy.

## Methods

### Animals

This study has been approved by the Experimental Animal Ethics Committee of Southwest Medical University. 6–8-week female rats weighted 180-200 g were purchased from Chongqing Tengxin Biotechnology Co., LTD., the laboratory animal certificate No. was SCXK(Liao)2020–0001. All rats were raised in SPF animal houses, with an indoor temperature of 22 + 2℃ and relative humidity of 40%-70%.

### Radiation pneumonitis rat model after interstitial brachytherapy

**Fig. 1:**
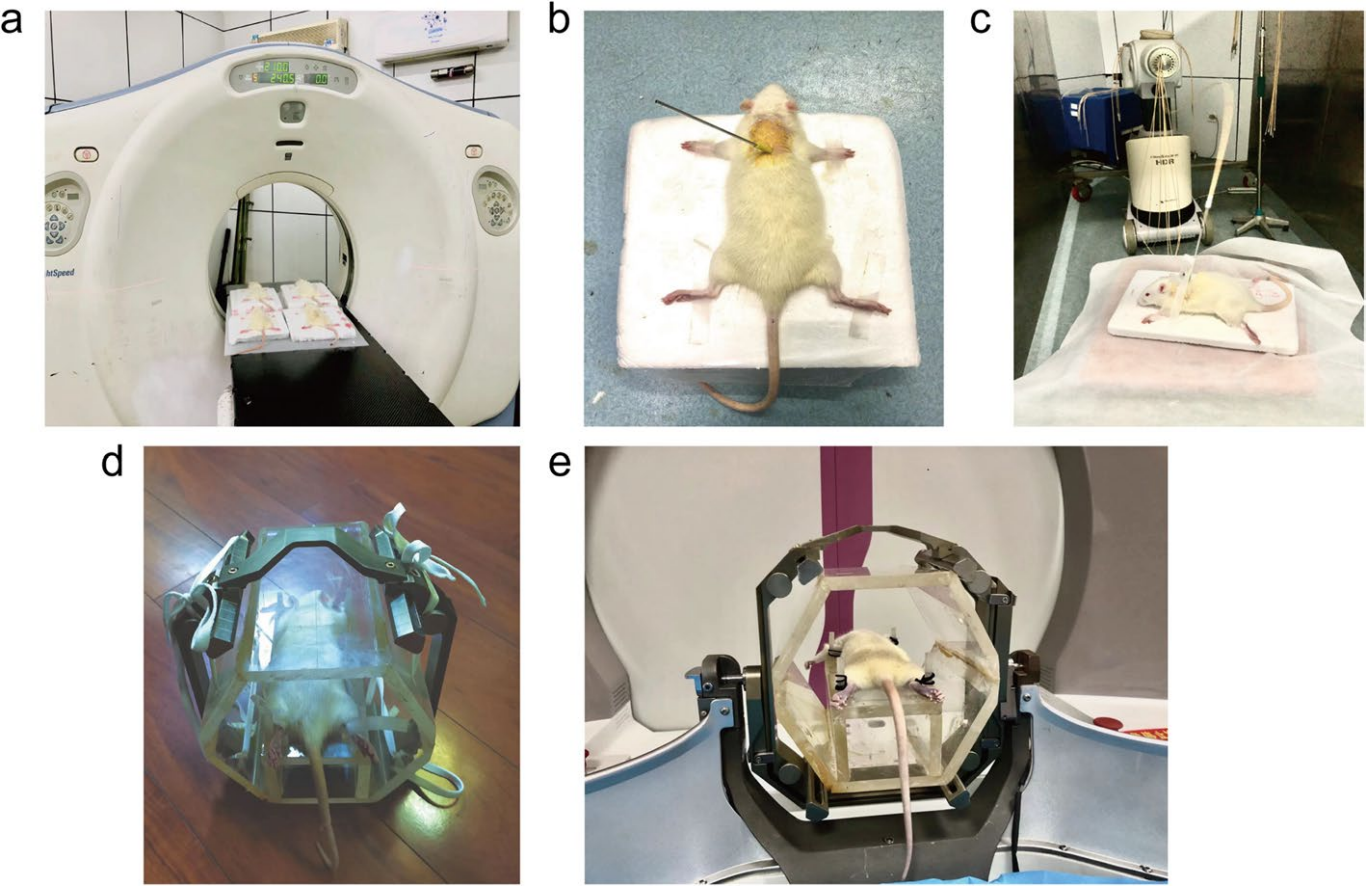
The schematic diagram of the establishment of RILI rat models. **a** CT localization in SD rats after anesthesia. **b** The needle was implanted in the right chest of SD rats under the guidance of CT. **c** Interstitial brachytherapy process. **d** Position fixator for SBRT in SD rats. **e** The procedure of SBRT

The radiation pneumonitis rat model after interstitial brachytherapy was established as follows. Briefly, the rats were anesthetized using an intraperitoneal chloral hydrate injection, followed by back hair removal and supine fixation on a special platform (Fig. 1). A spherical target with a diameter of 0.5 cm in the right middle lung was set, and both lungs were scanned by CT with a thickness of 0.2 cm. The obtained CT images were transferred to the radiotherapy planning software, and the CT image fusion, gross tumor volume (GTV) and organs at risk (OAR, including lungs, heart, esophagus, trachea, and spinal cord) were determined by the radiation oncologist. The prescribed dose of GTV with 90% was 30GY, and the dose limit of OAR was carefully applied based on the protocol. The simulated insertion needle was placed closest to the GTV, avoiding the ribs, blood vessels, heart, and other OARS. A manual/graphical optimization method of Oncentra 4.3 treatment planning software was applied to optimize the dose curve, ensuring that the curvilinear prescription dose around GTV and OAR received a lower dose. CT image fusion, GTV and OAR were outlined by the radiation oncologist. The sterilized needle was inserted into the right lung of the rats under the guidance of CT, and the right lung was irradiated with a single irradiation dose of 30GY using post-installation therapy machine with Ir192. After the treatment, the insertion needle and fixation device were removed, and the puncture point was disinfected with Iodophor. Chest CT was applied to reexamine the potential presence of pneumothorax, hemothorax, and hemoptysis. Meanwhile, the respiratory rate, heart rate, lung auscultation, activity, eating and hair condition were observed, as well as the lung radiation damage after irradiation, indicating that 30GY radiation dose could successfully induce radiation pneumonitis in rats during a short time.

### Radiation pneumonitis rat model after stereotactic body radiotherapy

**Fig. 2:**
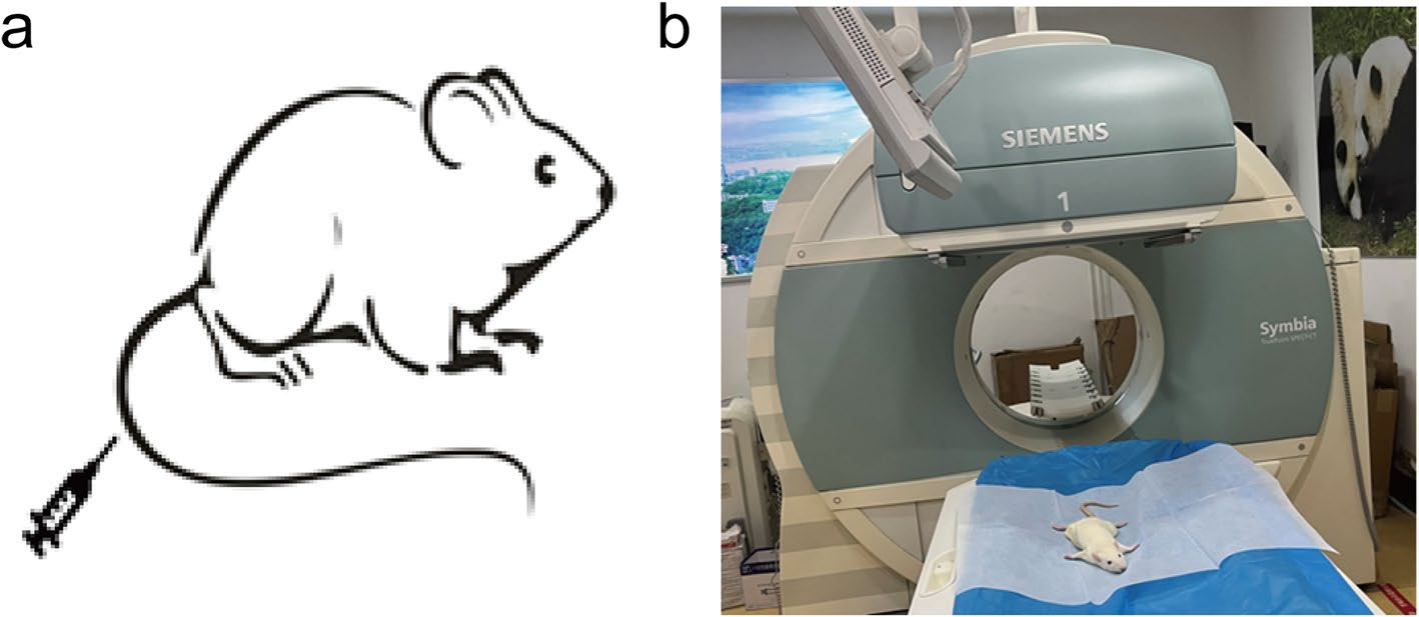
The procedure of SPECT/CT. **a** Technetium 99-labeled polymerized human serum albumin radioactive particles were injected through the rat tail vein. **b** SPECT/CT imaging was performed in SD rats to observe the uptake of technetium-99 polymerized human serum albumin in the lung tissue after injection of the tracer agent

The rats were anesthetized using an intraperitoneal chloral hydrate injection, followed by a supine fixation on a special platform (Fig. 2b). Similarly, the obtained MRI images were transferred to the radiotherapy planning software, and the MRI image fusion, GTV and OAR were determined by the radiation oncologist. The prescribed dose of GTV with 90% was 30GY, and the dose limit of OAR was carefully applied based on the protocol. A manual/graphical optimization method of SRRS + treatment planning software was applied to optimize the dose curve, ensuring that the curvilinear prescription dose around GTV and OAR received a lower dose. MRI image fusion, GTV and OAR were outlined by the radiation oncologist. The back skin of the rat was disinfected, and the rat was placed into the γ source stereotactic radiotherapy, with a single irradiation dose of 30GY in the right lung. After irradiation, the presence of conditions such as hemoptysis and dyspnea were observed.

### Animal grouping

A total of 72 healthy SD rats were randomly divided into group A, B and C with 24 rats per group. 4 rats in interstitial brachytherapy group (Group A) and stereotactic body radiotherapy group (SBRT, Group B) were randomly after 1, 4, 8 and 16 weeks, respectively. Group C: Control group. SD rats were placed into cages for further feeding after each treatment or examination.

### Monitoring the general condition of rats

The general condition including feeding, activity state, hair color, respiratory rate and response to external stimuli of rats were observed regularly every day. The body weight was measured and recorded once a week. The respiratory activity of the chest and abdomen of the rats was observed and recorded once a week when the rats were awake.

### Changes of radiation pneumonia evaluated by CT scan

Small animal micro-PET/CT was employed for low-resolution CT scanning in rats of different groups, 6 rats in each group fasted for more than 8 h in advance. The rats were anesthetized using an intraperitoneal chloral hydrate injection, then the rats were fixed in a micro-PET/CT scanner for CT scanning after 10-20 min of injection. The region of interest (ROI) in rats was plotted by two qualified nuclear medicine physicians, and the left and right lung volumes and CT values were calculated by a computer system.

### Rat lung function assessed by 99 m Tc-MAA SPECT/CT

To evaluate the pulmonary ventilation/blood perfusion of rats in interstitial brachytherapy and SBRT groups at the 16th week, respectively, SPECT/CT scan was applied to observe the uptake of technetium-99 polymerized human serum albumin in the lung tissue [20]. Briefly, the rats were anesthetized (Fig. 2a) and injected with technetium 99-labeled polymerized human serum albumin radioactive particles with a dose of 50-60uci through the tail vein, then the rat was placed in a prone position in SPECT/CT scanner for scanning (Fig. 2b). The ROI at each level of the rat lung was plotted by two qualified nuclear medicine physicians, and the lung perfusion of the left and right lungs, as well as the number of collected signals during SPECT/CT scan were analyzed.

### ELISA

5 ml of peripheral blood was collected and placed in an EP tube by aseptic eyeball blood collection method and naturally coagulated for 15 min at room temperature at 1, 4, 8 and 16 weeks after radiotherapy. Then the EP tube was centrifuged at 2000r/pm for 15 min, and the serum was collected and stored in an ultra-low temperature refrigerator. The serum concentrations of IL-2, INF-γ, IL-6, IL-10 and TGF-β of SD rats were detected based on the instructions of the ELISA kit.

### Pathological examination

The rat lung tissues at 1, 4, 8, and 16 weeks after treatment and examination were collected, and the procedure was shown as follows. Briefly, the chest of the rat was opened, and the full lungs were quickly and completely extracted. Lung tissues were rinsed in precooled normal saline to remove the blood, then the surface moisture of lung tissues was dried with filter paper. The lung tissues were put in 10% formaldehyde solution for 24 h for fixation, and the fixed lung tissues were dehydrated with an automatic dehydrator, followed by embedding and slicing. Each section was stained with hematoxylin and eosin (HE), and the images were observed and captured under the optical microscope. Images with ROI of 40 times and 400 times were recorded, and the infiltrating area and percentage of inflammatory cells in the right lung were observed under a 40 × field of view and quantified by Image-J software. The evaluation of HE images was independently conducted by two professionals.

### Statistical methods

SPSS18.0 software was applied for statistical analysis, the enumeration data were presented as mean ± standard error (χ + S). An Independent sample t-test was used to compare the data between the two groups. The body weight, respiratory rate, left and right lung volume, CT difference between left and right lung, lung ventilation/blood perfusion, and cytokines in each rat group were analyzed by One-way ANOVA. The statistical diagrams were plotted by using GraphPad Software Prism 9.0.

## Results

### The effect of interstitial brachytherapy on the general condition was less than SBRT in SD rats

**Fig. 3:**
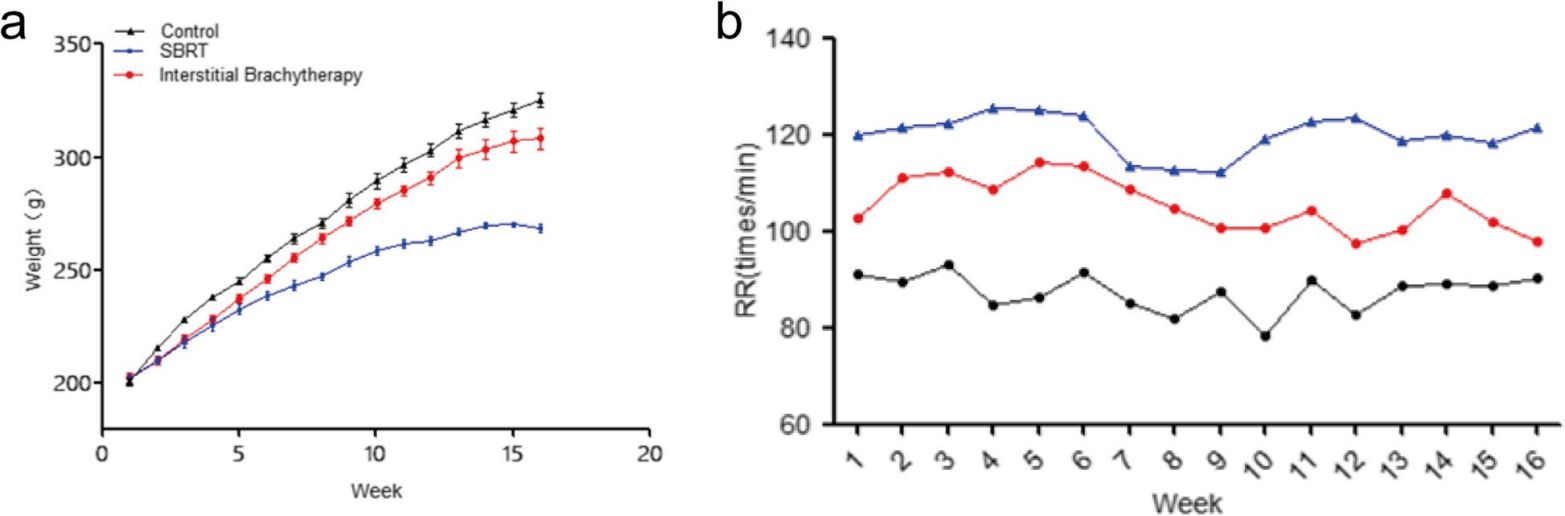
The alternations of the general condition in two treatment groups with time. **a** Changes in body weight of rats. **b** Changes of respiratory rate in rats

No obvious abnormal symptoms such as hemoptysis and dyspnea were observed in rats treated by both interstitial brachytherapy or SBRT. However, further manifestations including upturned fur, dullness, tight breath, decreased appetite, slow weight gain after 1 week of SBRT, and interstitial brachytherapy showed similar but slighter symptoms. As shown in Fig. 3a, the weight gain was slower in both interstitial brachytherapy and SBRT rat group when compared with the control group, while the increasing rate of interstitial brachytherapy was more rapid than SBRT rat group. Besides, the respiratory rate in both treatment groups was elevated (*P* < 0.05), in which interstitial brachytherapy was 108/min and SBRT was 125/min, and the respiratory rate in the control group was 85/min at 4 weeks post-treatment (Fig. 3b). Collectively, the SBRT had more obvious effects on body weight, respiratory rate and general condition of rats.

### The difference in lung volume and CT value of the left and right lungs in SD rats

**Fig. 4:**
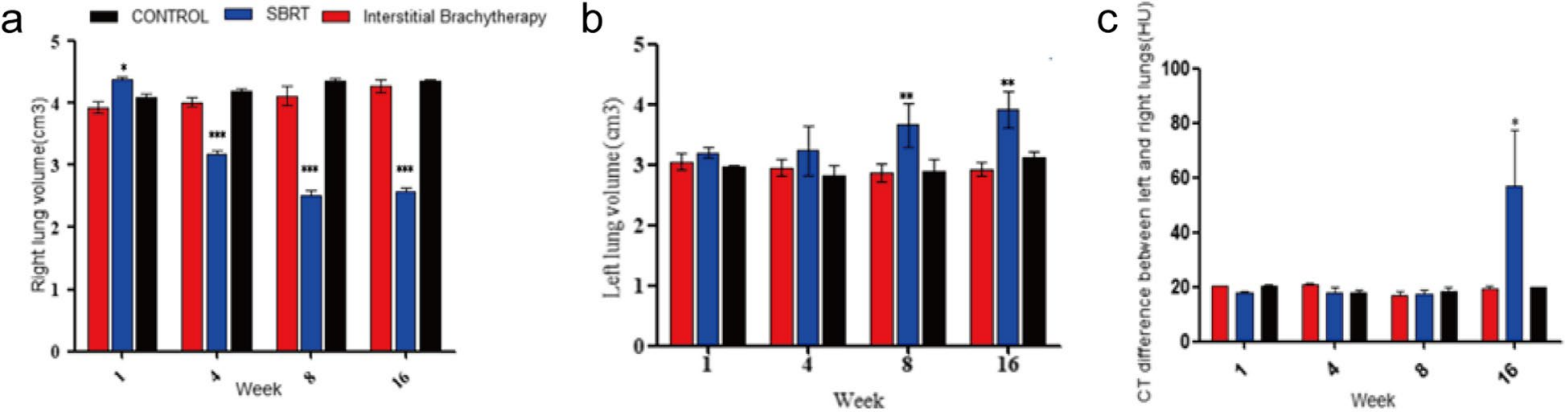
The difference of lung volume and CT value of the left and right lungs in SD rats. **a** Right lung volume checked by chest CT every 4 weeks. **b** Comparison of left lung volume among three groups. **c** Comparison of mean CT difference between left and right lungs of rats in three groups. *P < 0.05, **P < 0.01, *** *P* < 0.001

To further observe the presence of radiation pneumonia, SD rats were anesthetized and underwent chest CT scans at 1, 4, 8 and 16 weeks, respectively. The results showed that the right lung volume of SBRT group was larger than that of interstitial brachytherapy group and control group. However, the right lung volume of SBRT group began to reduce and maintain at the same volume in the first week, which is significantly smaller than interstitial brachytherapy rat group (*P* < 0.05). As time goes on, no significant change in right lung volume was observed between control group and the interstitial brachytherapy group (*P* > 0.05). At the 16th week, the right lung volume in the control group was 4.38cm^3^, 4.27cm^3^ in the interstitial brachytherapy group and 2.56cm^3^ in the SBRT group (Fig. 4a). On the contrary, the left lung volume in the SBRT group began to increase over time, and it was larger than 3cm^3^ in the control group (*P* < 0.05) (Fig. 4b); besides, left lung volume in the SBRT group was significantly higher than interstitial brachytherapy group at the 8th week (*P* < 0.05). There was no significant difference in the left lung volume between the control and the interstitial brachytherapy group (*P* > 0.05).

To identify the tissue types such as fat, effusion or calcification represented in CT, we compared the difference between the right and left lung in three rat groups. The results showed that at the 16th week, the differences between right and left lung CT values were significantly elevated in SBRT group when compared with the control group and the interstitial brachytherapy group (*P* < 0.05) (Fig. 4c). However, no significant difference of CT values between interstitial brachytherapy and SBRT groups was noticed. These results indicated that SBRT has a certain influence on lung tissue density in rats.

### Pathological examination among interstitial brachytherapy, SBRT and control rats

**Fig. 5:**
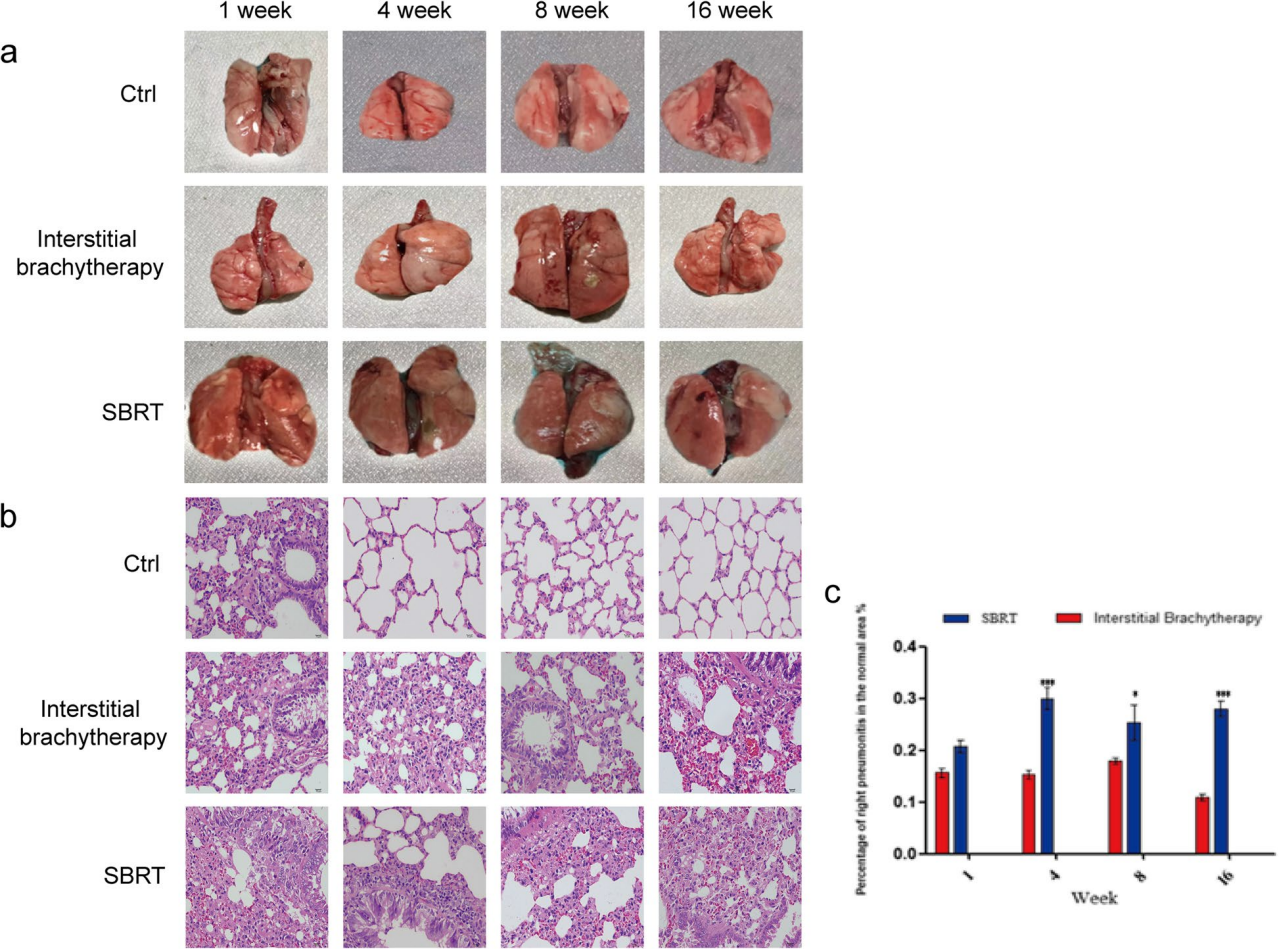
Overall view, microscopic view and percentage of the inflammation area in the normal area of the lung in the three groups. **a** Overall view of lung tissues in control, interstitial brachytherapy and SBRT groups. **b** Microscopic view of lung tissues in control, interstitial brachytherapy and SBRT groups, × 400. **c** Percentage of the inflammation area in control, interstitial brachytherapy and SBRT groups every 4 weeks. *P < 0.05, **P < 0.01, *** *P* < 0.001

After 1, 4, 8 and 16 weeks of radiotherapy, the gross anatomy of lungs in different rat groups was observed. It was shown that lung tissue in control group was smooth, soft and pink, without congestion and edema. While at the 8th week of the interstitial brachytherapy group, the color of lung tissue was deepened and slightly swollen, besides, a fibrous sclerotic lesion with a radius of about 0.2 cm was observed in the right lower lung. At the 16th week, mild atrophy was observed in the right lung without obvious changes in the shape of the whole lung. In the SBRT group, we found that the color of the right lung was deepened 1 week after treatment, accompanied by visible petechiae in the right middle lung; there were gray and white spots in the irradiation site of the right lung at the 8th week, and the lung tissue was shown as dark red with obvious swelling; at the 16th week, the volume of the right lung was firmly reduced and hard, whereas the volume of the left lung was increased with dark red color (Fig. 5a).

The 40 × imaging results of HE staining were represented in Fig. 5b, the lung tissue structure in the control rat group was normal, the bronchial ciliary epithelial cells were orderly arranged, and no exfoliated cells were observed, the alveolar epithelial cells were normal and full without obvious degeneration and necrosis, no proliferation of fibrous tissue and inflammatory cell infiltration in the lung interstitial, and no other special pathological changes were noticed. In the interstitial brachytherapy group, the lung tissue structure was relatively normal, but there was thickened alveolar septum at varying degrees, accompanied by alveolar epithelium and fibrous tissue proliferation, no obvious pathological changes were observed in other structures. In the SBRT group, the alveolar septum was obviously thickened, and the blood vessels were reduced. There were more fibrocyte and epithelial cell proliferation, and some macrophages and foam cells have been detected in the proliferative area.

We further performed the inflammatory infiltration analysis at a magnificent 400 × by Image J software. The results showed that the percentage of right pneumonitis in the normal area in the SBRT group was slightly larger than that of interstitial brachytherapy group without a significant difference at week 1 (*P* > 0.05). However, the difference became significant at week 4, 8 and 16 (*P* < 0.05). The pneumonitis lesion area in the SBRT group gradually increased with time, and was stable at 23%, while the area in the interstitial brachytherapy group remained stable at 15%. Thus, we indicated that the degree of lung injury was more severe after SBRT, and the degree of lung injury of these two types of radiation did not gradually ameliorate over time (Fig. 5c).

### Serum cytokines among interstitial brachytherapy, SBRT and control rats

**Fig. 6:**
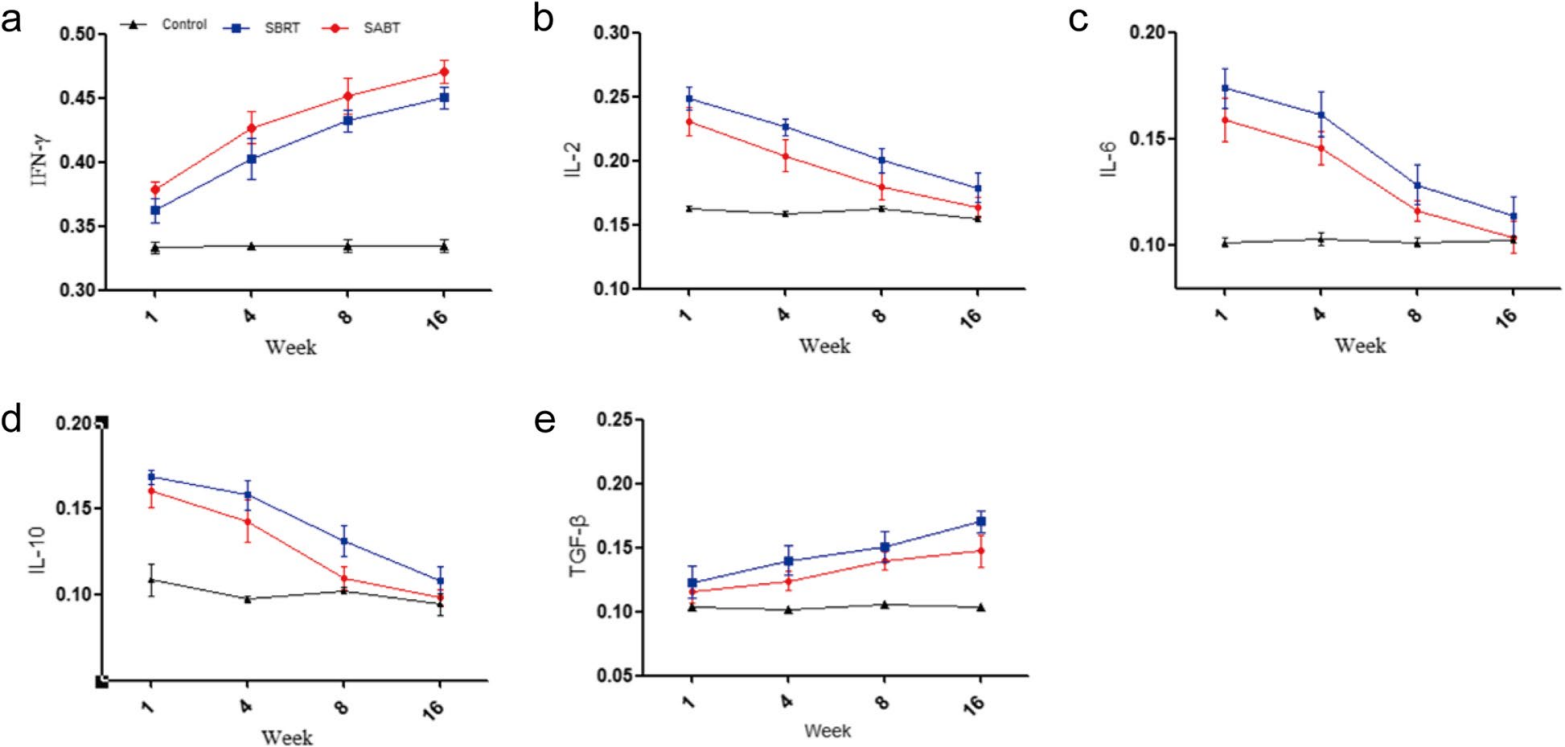
The concentration changes of inflammatory cytokines, antifibrotic cytokines and profibrotic cytokines in rat serum were detected by ELISA. The serum IFN-γ (**a**), IL-2 (**b**), IL-6 (**c**), IL-10 (**d**) and TGF-β (**e**) expressions (pg/ml) in each group were measured by ELISA every 4 weeks. *P < 0.05, **P < 0.01, *** *P* < 0.001

As shown in Fig. 6a, the IFN-γ expression was increased with time in the interstitial brachytherapy group, and reached its peak at week 16. The IFN-γ expression in interstitial brachytherapy group was significantly different from that in the SBRT group at week 1, 4, 8 and 16 (*P* < 0.05). Besides, the expressions of IL-2, IL-6 and IL-10 in SBRT group were significantly higher than that of interstitial brachytherapy group, and they represented a declining tendency with time (Fig. 6b-d). As shown in Fig. 6e, the TGF-β expression in interstitial brachytherapy group reached its peak with the increase of time from week 1 to week 16, and it was significantly lower than SBRT group (*P* < 0.05), indicating the presence of more fibrosis-promoting cytokines and heavier pulmonary fibrosis in rats of SBRT group.

### The effect of interstitial brachytherapy and SBRT on pulmonary ventilation/perfusion

**Fig. 7:**
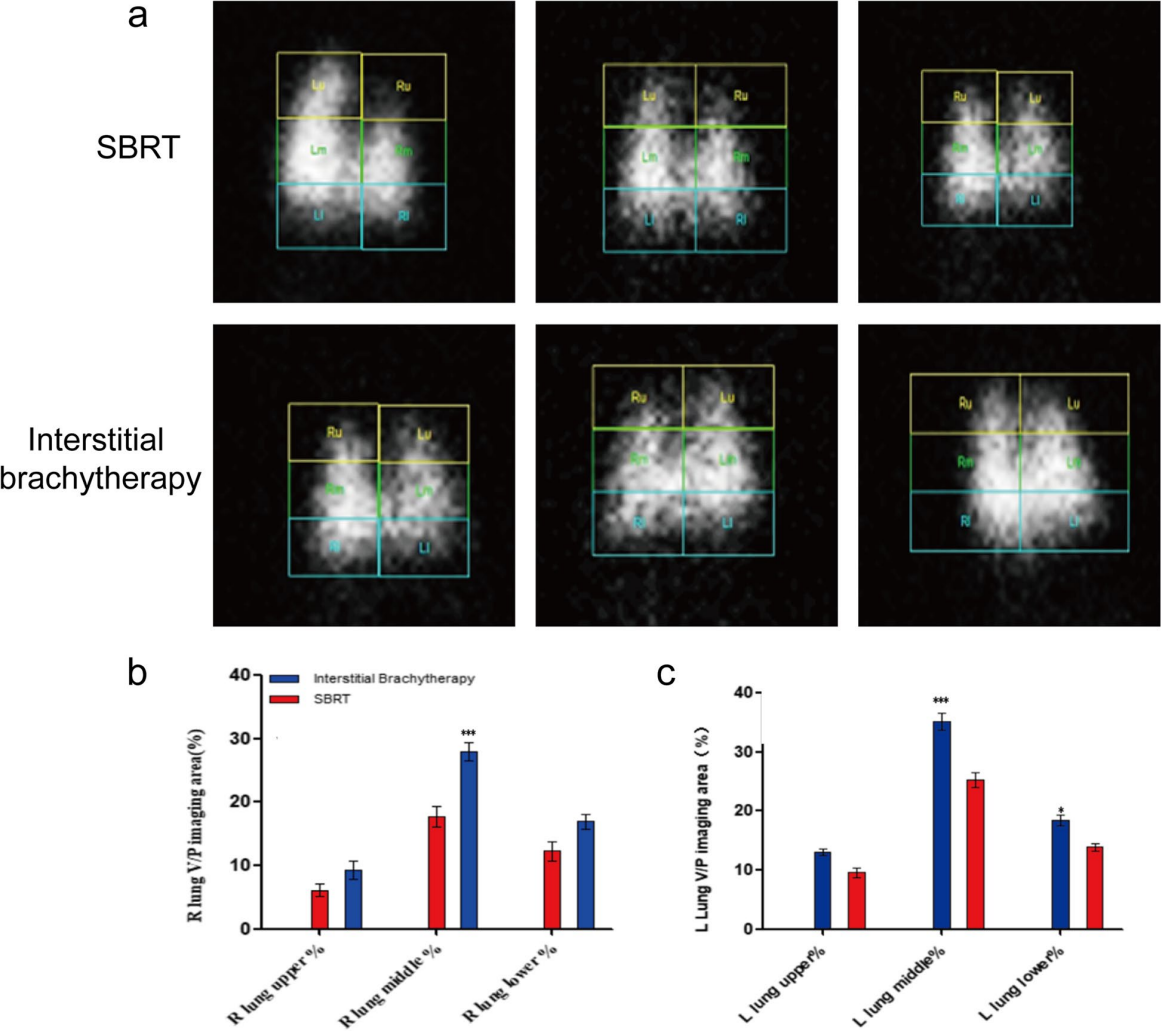
Lung perfusion imaging, right lung perfusion and left lung perfusion examinations. **a** Pulmonary perfusion imaging of SD rats at 16 weeks in interstitial brachytherapy and SBRT groups. Right (**b**) and left lung V/P imaging area (%) (**c**) in interstitial brachytherapy and SBRT groups. *P < 0.05, **P < 0.01, *** *P* < 0.001

CT perfusion imaging was conducted to assess the impact of two treatment approaches on pulmonary ventilation/perfusion (V/P). As shown in Fig. 7a, the V/P of the right lung in SBRT group was poor, while the V/P of the left lung was better. In the interstitial brachytherapy group, no obvious difference of V/P in the right and left lung was noticed. Further quantitative SPECT/CT results suggested that the V/P imaging area (%) of right middle lung was significantly larger in interstitial brachytherapy group when compared with SBRT group, while the V/P imaging area (%) of left middle and lower lung was significantly smaller in the SBRT group compared with interstitial brachytherapy group (all *P* < 0.05) (Fig. 7b, c).

### The difference in mortality in interstitial brachytherapy and SBRT rat groups

**Fig. 8:**
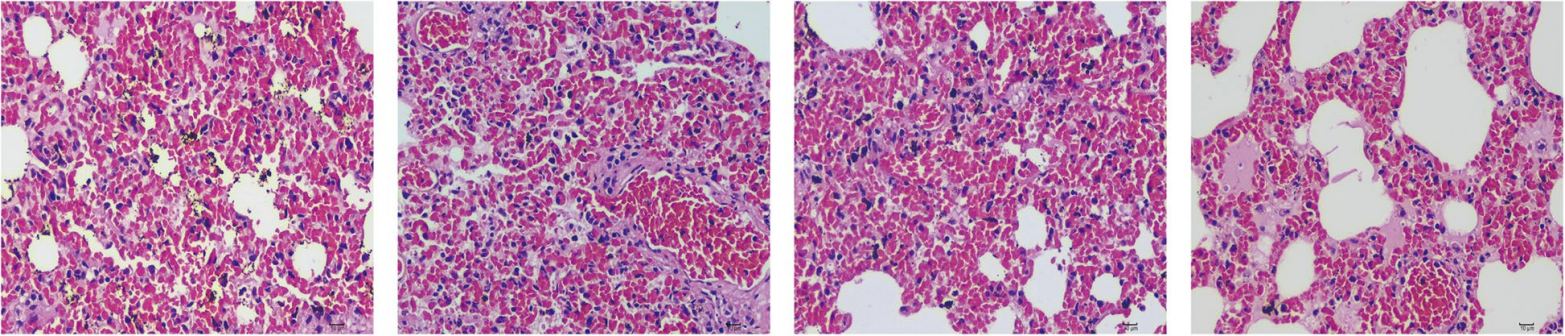
Pathological sections of dead rats

After the two different treatments, we found the presence of fatal acute radiation pneumonia developed in 3.2 days after SBRT, manifesting crouching, hair erection and loss, listlessness, shortness of breath, food refusal, reduced activity, and death, with a mortality rate of 16.7% (Table 1). Pathological biopsy was performed immediately, as shown in Fig. 8, a large amount of blood congestion was observed in pulmonary vessels and interstitial under a light microscope, accompanied by the thickened alveolar septum, alveolar epithelial cells and elastic fibers proliferation, and inflammatory cells infiltration such as lymphocytes and neutrophils. A small amount of protein-like substance exudates in the lung interstitial. There were no dead rats in the control group and interstitial radiotherapy group.

**Table 1:** Comparison of mortality rate between two groups (n, %)

Group	n	Dead rats (n)	Mortality rate (%)
Control group	24	0	0
Interstitial brachytherapy group	24	0	0
SBRT group	24	4	0.167

## Discussion

During radiotherapy for malignant tumors such as lung cancer, breast cancer, esophageal cancer, and thymic cancer, normal lung tissue around the target area will inevitably be exposed to the rays, thus leading to damage with varying degrees to normal lung tissue, which may sometimes even threaten the life safety of patients [21, 22]. The alveolar-capillary complex is the most sensitive subunit during radiotherapy for lung tumor patients, and RILI occurs in more than 50% of patients receiving SBRT [23, 24]. In this study, we evaluated the effects of two radiotherapy methods including interstitial brachytherapy and SBRT on the lung volume and the percentage of lung tissue lesions in normal tissues by using CT images, and the results indicated that the interstitial brachytherapy had fewer systemic side effects, the degree of pulmonary fibrosis was less in the late stage, which showed a better protective role than SBRT on normal lung tissues. In addition, the serum levels of inflammatory cytokines and fibrotic cytokines in interstitial brachytherapy were significantly lower than SBRT group, indicating the pulmonary toxicity of interstitial brachytherapy was less, with a slighter impact on lung function. To the best of our knowledge, this is the first study to compare the effects of interstitial brachytherapy and SBRT on lung injury, and our results underscore that interstitial brachytherapy is safer with less injury on lung tissues when compared with SBRT rat group. These novel findings help select the optimal therapeutic approach for patients with advanced metastasis lung cancer, achieving similar outcomes more safely.

Over decades, radiotherapy has undergone a paradigm shift in strategy and delivery, successfully reducing the incidence of RILI. The clinical manifestations of RILI include cough, decreased basic lung function, shortness of breath, and increased respiratory rate, the abnormal alternations could also be found in auscultation and imaging examinations [25, 26]. Radiation pneumonia is usually diagnosed by exclusion, medicines including steroids, ACE inhibitors and theophylline have formed the cornerstone of treatment [27]. Growing evidence showed that radiation-related pneumonia and fibrosis may be the results of the interaction between chronic inflammation and oxidative damage [28]. The activation of alveolar macrophages plays an important role in RILI, which is largely responsible for the persistent cytokine cascade and chronic inflammatory state involving early and late RILI [29]. The specific underlying mechanism of RILI remains elusive.

In recent years, immune cells and inflammatory cytokines have sparked widespread enthusiasm in the RILI research field [30, 31]. IL-2, IL-6 and IL-10 belong to inflammatory cytokines, they can promote inflammatory response through upregulating chemokines, stimulating the secretion of cytokines by various immune cells, inducing acute phase reaction proteins, and coordinating with intracellular signal transduction [32–34]. IFN-γ, mainly produced by T lymphocytes, could downregulate collagen and resist fibrosis by inhibiting IL-4 and IL-13 [35, 36]. After exposure to radiotherapy, the involved lung lesion produces a high level of TGF-β, which is considered as an important marker of fibrosis in RILI at a late stage [37–39]. In the current study, inflammatory cytokines and profibrotic cytokines were detected to assess the RILI degree in different therapeutic rat groups including interstitial brachytherapy and SBRT. Our findings suggested that the production of inflammatory cytokines and profibrotic cytokines was elevated in the SBRT group, contributing to acute radiation pneumonia and severe pulmonary fibrosis in the late stage of radiotherapy. Therefore, it is urgent to select an optimal alternation with less lung toxicity in lung cancer treatment. Moreover, we also found that the expressions of IL-2, IL-6 and IL-10 were increased to a high level at the early stage, and then decreased to a normal level at the 16th week, while the expression of TGF-β and IFN-γ continued to maintain at a high level with time, suggesting that the clinical treatment of RILI should be extended to 4 months after the end of treatment, and attention should be paid to different treatment priorities at different times.

Chest CT is a common clinical tool for RILI examination, which can screen the alternation of RILI in advance [40]. Although the CT findings of acute RILI are not very obvious, advanced RILI may result in increased interstitial density and reduced lung volume. Here, we noticed the difference in the injury degrees of interstitial brachytherapy and SBRT with time. At 1-week post-treatment, right lung volume in SBRT was larger than interstitial brachytherapy group shown by CT images, while no significant pathological changes were observed between interstitial brachytherapy and control group. We hypothesized that the less right lung volume in interstitial brachytherapy rats may be caused by the limited breathing movement induced by pain. As time went by, the volume of the irradiated right lung in SBRT group was significantly smaller than that of the interstitial brachytherapy group.

Since chest CT can only visualize morphological features of radiation pneumonia, we further performed the lung perfusion at the 16th week to assess the two radiotherapy methods on lung V/P. The results confirmed that the right lung V/P in the interstitial brachytherapy group was better than SBRT group, whereas the left lung V/P in the SBRT group was better than interstitial brachytherapy group. We speculated that the right lung ventilation might be ineffective due to advanced pulmonary fibrosis in SBRT group, accompanied by pulmonary hypertension and reduced respiratory function in rats. Meanwhile, the left lung was compensated due to more severe injury and poor ventilation/perfusion of right lung in the SBRT group, these findings are in line with the study reported by Z. Vujaskovic [41].

There are some limitations in the present study. First, since the occurrence and development process of RILI is complicated, we did not further explore the underlying molecular mechanism triggered by two different radiotherapy methods over time. Second, we did not distinguish between pulmonary inflammation and pulmonary fibrosis, further study with a detailed classification of various pathological changes is needed. Last, further studies with larger experimental sample sizes and longer experimental periods to observe the alternative results of RILI and survival analysis are required.

## Conclusion

Collectively, this study confirmed the relatively safe features of interstitial brachytherapy with similar curative effects compared with SBRT. Interstitial brachytherapy had fewer systemic side effects and a better protective role than SBRT on normal lung tissues. Besides, the serum levels of inflammatory cytokines and fibrotic cytokines in interstitial brachytherapy were significantly lower than SBRT group. Therefore, the therapeutic way of interstitial brachytherapy is expected to elevate the tumor control rate, prolong the survival period, and improve the quality of life by reducing the side effects of radiotherapy and increasing the radiation dose of radiotherapy.

## Data Availability

The data that support the findings of this study are available from the corresponding author upon reasonable request.

## Abbreviations

RILI: Radiation-induced lung injury
SBRT: Stereotactic radiotherapy
IL: Interleukin
IFN: Interferon
TGF: Transforming growth factor
GTV: Gross tumor volume
