# Editorial Expression of Concern: The occurrence and development of radiation-induced lung injury after interstitial brachytherapy and stereotactic radiotherapy in SD rats

**DOI:** 10.1186/s12950-026-00506-9

**Published:** 2026-06-08

**Authors:** Zhuo Chen, Bin Wang, Zhouxue Wu, Hua Xiao, Yang Yang, Junying Fan, Yingjiang Gu, Chuan Chen, Jingbo Wu

**Affiliations:** 1 Department of Oncology, Daping Hospital, Army Medical University, 10 Changjiang Branch Road, Yuzhong District, Chongqing, 400042 China; 2 Department of Oncology, Affiliated Hospital of Southwest Medical University, No.25 Taiping Street, Jiangyang District, Luzhou, Sichuan 646099 China; 3 Department of Oncology, the Seventh People’s Hospital of Chongqing (Affiliated Central Hospital of Chongqing University of Technology), Banan District Lijiatuo Industry Federation No.1 Village, Chongqing, 401320 China; 4 Department of Neurosurgery, Affiliated Hospital of Traditional Chinese Medicine of Southwest Medical University, Longmatan District, No. 182 Chunhui Road, Luzhou, Sichuan 646099 China; 5 Key Laboratory of Nuclear Medicine and Molecular Imaging, Changzhi, Sichuan 046099 China

**Editorial Expression of Concern: Journal of Inflammation (2023) 20:23**

10.1186/s12950-023-00348-9

The Editor-in-Chief is issuing an editorial expression of concern to alert readers that after the publication of this article, it was brought to the attention of the publisher that concerns were raised with Fig. 7, additional editing (beautification) was found with Fig. 5. The authors were able to provide some original data, but not all of the original data upon request, as stated in the article, for validation and to ensure replicability. Readers are urged to take caution when interpreting the content and conclusions of this article.

Author Zhuo Chen agrees with this Editorial Expression of Concern. All remaining authors did not respond to correspondence from the Publisher about this Editorial Expression of Concern.

